# Carotid Artery Stenting Versus Carotid Artery Endarterectomy in Asymptomatic Severe Carotid Stenosis: An Updated Meta-Analysis

**DOI:** 10.7759/cureus.50506

**Published:** 2023-12-14

**Authors:** Ankita Aggarwal, Cameron Whitler, Anubhav Jain, Harshil Patel, Marcel Zughaib

**Affiliations:** 1 Cardiology, Ascension Providence Hospital - Southfield Campus, Southfield, USA; 2 Cardiology, Ascension Genesys Hospital, Grand Blanc, USA

**Keywords:** stroke, carotid endarterectomy (cea), carotid artery stenosis, carotid stent, carotid angioplasty

## Abstract

Carotid artery stenting (CAS) and carotid artery endarterectomy (CEA) are revascularization options for the management of severe carotid disease in asymptomatic patients. We aimed to compare the peri-procedural outcomes of the two modalities. A systematic review of the databases PUBMED, EBSCO, and Cochrane Library was performed. All the studies that reported periprocedural outcomes (within 30 days) in asymptomatic carotid stenosis patients were included in the meta-analysis. Random effects models with inverse-variance weighting were used to estimate pooled risk ratios (RRs) to compare the outcomes. Fifteen studies (including seven randomized controlled trials) met the inclusion criteria. A total of 15251 patients were included, out of which 6419 (42%) underwent CAS and 8832 (57.9%) underwent CEA. There was no statistical difference in the primary composite outcome of death/stroke/myocardial infarction (MI) (RR 1.02, 95% CI [0.69-1.51], p 0.93). No difference was found in the secondary outcome of all-cause mortality. CAS was associated with a slightly lower risk of MI and cranial nerve palsy. CAS was associated with a slightly higher risk of stroke with no difference in the occurrence of disabling stroke or ipsilateral stroke. In general terms, the study confirms equipoise in the two treatment strategies with a higher risk of MI and cranial nerve palsy with CEA and a higher risk of non-disabling stroke with CAS.

## Introduction and background

Stroke is one of the leading causes of mortality and morbidity in the United States and is associated with high morbidity [1]. About 15% of strokes are caused by atherosclerosis of the extracranial carotid artery [2]. Asymptomatic carotid artery stenosis (ACAS) is defined as a ≥50% narrowing of the carotid artery in the absence of retinal or cerebral ischemia in the preceding six months. Based on age and gender, the prevalence of asymptomatic carotid stenosis ranges from 0.5% to 7% in the general population [3]. A recent meta-analysis evaluating the risk of stroke in this population found the incidence of stroke to be as high as 4.3% in patients with high-risk plaque features [4]. Revascularization with either carotid artery endarterectomy (CEA) or carotid artery stenting (CAS) plays an important role in stroke prevention. The most recent 2011 American College of Cardiology/American Heart Association (ACC/AHA) Guideline on Management of Patients with Extracranial Carotid and Vertebral Artery Disease recommends CEA as first-line therapy, with CAS as a reasonable alternative in symptomatic patients [5]. This consensus guideline also states that it is reasonable to perform revascularization in asymptomatic patients who have more than 70% stenosis of the internal carotid artery (ICA) if the risk of perioperative stroke, myocardial infarction (MI), and death is low. They recommend choosing CEA in older patients and CAS when CEA cannot be performed due to unfavorable anatomy [5]. Similarly, the 2017 European Society of Cardiology guidelines recommend CEA to be performed in asymptomatic patients with an increased risk of stroke if the perioperative risk is <3% [6]. Further, they recommend CAS in high-risk (Class IIa) and average-risk patients (Class IIb) when CEA cannot be performed [6].

In recent years, CAS has been proposed as a safer alternative due to the lower risk of cranial nerve palsy and cardiovascular complications. Nevertheless, there is a concern for an increased risk of perioperative stroke with CAS [7]. However, with further advancements including the introduction of embolic protection devices, this risk appears to be significantly reduced. A study by Kastrup et al. showed that the use of an embolic protection device during CAS led to a statistically significant reduction in the occurrence of both major and minor strokes from 1.1% to 0.3% and from 3.7% to 0.5%, respectively [8]. A recent meta-analysis comparing the two modalities showed a lower risk of MI but a non-significant trend toward an increased risk of periprocedural stroke with CAS [9]. Since the publication of this meta-analysis, new data has emerged comparing the safety and efficacy of CAS with CEA. Due to conflicting literature and the emergence of new data in the field, we aimed to perform an updated systematic review and meta-analysis comparing the periprocedural outcomes of the two modalities for the management of asymptomatic severe carotid stenosis.

The preliminary results of our meta-analysis were presented at the 2022 Society for Cardiovascular Angiography & Interventions (SCAI) Annual Meeting on May 19, 2022.

## Review

Materials and methods

Data Sources and Search Strategy

The recent Preferred Reporting Items for Systematic Reviews and Meta-Analyses (PRISMA) guidelines were followed to design the study [10]. Electronic databases including PubMed, EBSCO, and Cochrane Central Register of Controlled Trials (CENTRAL), were systematically searched using a combination of standardized medical subject headings (MeSH) and keywords including "carotid stenosis", "carotid endarterectomy”, and “carotid stenting”. The search items were combined using Boolean operators (“OR” or “AND”). No filters including publication year, publication design, or language were applied. In addition, reference lists of all retrieved studies were searched manually for potentially relevant studies not found in the initial search.

Study Selection and Data Extraction

Two independent authors initially screened studies using the title and abstract followed by reading the full text of the selected articles for data extraction. Disagreements were resolved by consensus. We included full-length articles published in the English language that compared outcomes of CAS and CEA in asymptomatic patients with severe carotid artery stenosis. All extracted data from the included studies were collected into a spreadsheet and verified by a third author. Data regarding baseline characteristics including mean age, gender, and presence of comorbidities outcomes were collected. The primary outcome of interest was a composite of periprocedural (within 30 days of index procedure) death/any stroke/MI. Secondary outcomes included all-cause mortality, any stroke, disabling stroke, ipsilateral stroke, MI, and cranial nerve palsy. Quality assessment was performed by two independent reviewers using the Cochrane Risk of Bias Tool and New Castle Ottawa (NOS) scale [11,12].

Statistical Analysis

The statistical analysis was performed using the Cochran-Mantel-Haenszel method under the random-effects model to calculate the risk ratio using DerSimonian and Laird random effects model [13,14]. The estimated effect size was reported as a point estimate and 95% confidence interval (CI). An alpha criterion of a p-value < 0.05 was considered statistically significant. Higgins's I-squared (I2) statistical model was used to evaluate variations in the outcomes of included studies [15]. I2 values of 25%, 50%, and > 75% indicated low, moderate, and large amounts of heterogeneity, respectively. A sensitivity analysis was done using a fixed effect model. All statistical analysis was performed using the Cochrane Review Manager (RevMan v5.3, The Cochrane Collaboration, Oxford, UK) [16].

Results

**Figure 1:**
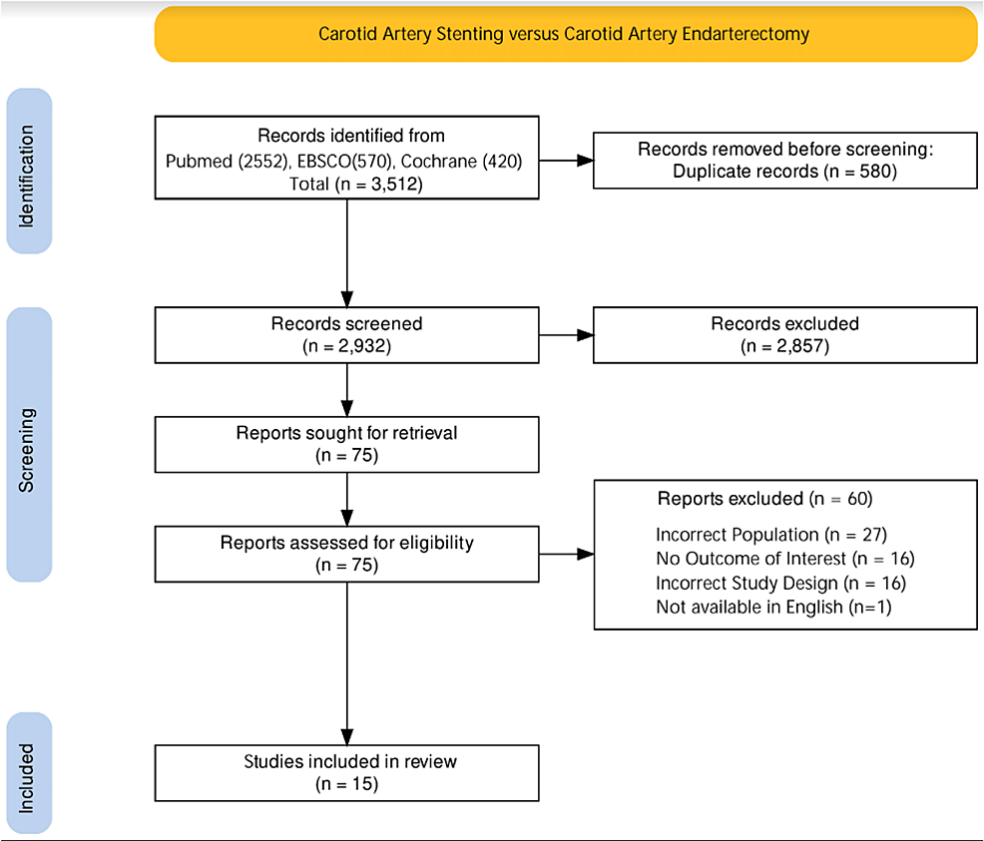
Search strategy and study selection per PRISMA flow diagram. PRISMA: Preferred Reporting Items for Systematic Reviews and Meta-Analyses

A total of 3512 articles were identified after our initial search. After the removal of duplicates (n=580), the articles were screened using their titles and abstracts. Seventy-five articles were deemed relevant for full-text review after the initial screening. Based on inclusion criteria, a total of 17 studies were found eligible. Out of these, one study was excluded as the full manuscript was not available in English, and one study was excluded due to a lack of reporting of any primary or secondary outcomes pertinent to the analysis in asymptomatic patients (Figure 1) [17,18].

Thus, a total of 15 studies comprising seven randomized controlled trials [19-25], five retrospective studies [26-30], one study with combined prospective and retrospective data [31], and two prospective non-randomized studies [32,33] were included in the final analysis. Table 1 summarizes the characteristics of these studies.

**Table 1:** Characteristics of included studies RCT: randomized controlled trial, CAS: carotid artery stenting, CEA: carotid artery endarterectomy

	Study	Year	Type of Study	Country	Number of Patients (Asymptomatic)		
					Total	CAS	CEA
1	ACST2 [19]	2021	RCT	Multiple	3625	1811	1814
2	SPACE 2 [20]	2019	RCT	Europe	400	197	203
3	Mannheim et al. [21]	2017	RCT	Israel	136	68	68
4	Meller et al. [26]	2016	Retrospective	USA	448	105	343
5	Brooks et al. [22]	2003	RCT	USA	85	43	42
6	ACT 1 [23]	2016	RCT	USA	1453	1089	364
7	Brown et al. [27]	2008	Retrospective	USA	129	79	50
8	Bradac et al. [28]	2014	Retro	Czech Republic	747	419	328
9	CaRess [32]	2009	Prospective	USA	133	99	170
10	Akinci et al. [29]	2016	Retro	Turkey	114	20	94
11	Kastrup et al. [31]	2003	Retro/Prospective	Germany	87	37	50
12	Ferreira et al. [30]	2019	Retrospective	Portugal	4695	854	3841
13	SAPPHIRE [25]	2004	RCT	USA	237	117	120
14	CREST [24]	2010	RCT	USA & Canada	1181	594	587
15	Yang et al. [33]	2020	Prospective	China	1645	887	758

Quality Assessment

**Figure 2:**
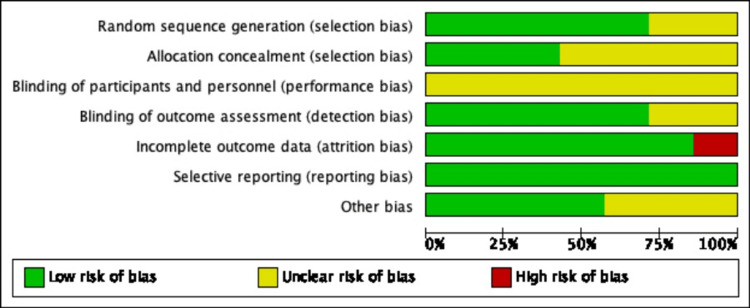
Revised Cochrane Collaboration’s tool for quality assessment in trials comparing CAS to CEA. CAS: carotid artery stenting, CEA: carotid artery endarterectomy

Cochrane Collaboration’s tool and NOS scale were used to assess the risk of bias and quality of randomized controlled trials (RCTs) and observational studies respectively. The overall risk of bias was found to be low. On visual assessment, there was no significant asymmetry noted on the funnel plot indicating a low risk of publication bias (Figure 2).

Baseline Characteristics

A total of 15251 patients were included, out of which 6419 (42%) underwent CAS and 8832 (57.9%) underwent CEA. In the pooled analysis, the baseline characteristics of the two intervention groups were found to be similar (Table 2). The proportion of females was 29.2% (CAS) and 26.3% (CEA). The prevalence of comorbidities like diabetes (31.5% vs 31.1%), hypertension (65.3% vs 68.3%), dyslipidemia (54% vs 54.5%), and smoking (45.4% vs 37.2%) were similar in the CAS vs CEA groups.

**Table 2:** Baseline characteristics of the study population RCT: randomized controlled trial, CAS: carotid artery stenting, CEA: carotid artery endarterectomy, DM: diabetes mellitus, HTN: hypertension

No.	Study	Year	Type of Study	Total Number of Patients	CAS (Number of Patients)	CEA (Number of Patients)	Mean Age (CAS)	Mean Age (CEA)	Number of Women (CAS)	Number of Women (CEA)	DM (CAS)	DM (CEA)	HTN (CAS)	HTN (CEA)	Dyslipidemia (CAS)	Dyslipidemia (CEA)	Smokers (CAS)	Smokers (CEA)
1	ACST2	2021	RCT	3625	1811	1814	N/A	N/A	539	541	542	543	668	658	682	708	N/A	N/A
2	SPACE 2	2019	RCT	400	197	203	70	70	54	52	59	52	177	180	158	158	99	91
3	Mannheim	2017	RCT	136	68	68	69	68	23	20	32	33	58	57	56	50	15	20
4	Meller	2016	Retrospective	448	105	343	71	72	N/A	N/A	N/A	N/A	N/A	N/A	N/A	N/A	N/A	N/A
5	Brooks	2003	RCT	85	43	42	66	69	N/A	N/A	7	5	35	41	9	8	40	37
6	ACT 1	2016	RCT	1453	1089	364	67	67	423	157	388	118	987	326	980	320	803	259
7	Brown	2008	Retrospective	129	79	50	70	67	N/A	N/A	N/A	N/A	N/A	N/A	N/A	N/A	N/A	N/A
8	Bradac	2014	Retro	747	419	328	N/A	N/A	N/A	N/A	N/A	N/A	N/A	N/A	N/A	N/A	N/A	N/A
9	CaRess	2009	Pros	269	99	170	72.2	71.9	40	60	31	41	82	141	68	124	N/A	N/A
10	Akinci	2016	Retro	114	20	94	N/A	N/A	N/A	N/A	N/A	N/A	N/A	N/A	N/A	N/A	N/A	N/A
11	Kastrup	2003	Retro/pros	87	37	50	n/a	n/a	n/a	n/a	n/a	n/a	n/a	n/a	n/a	n/a	n/a	n/a
12	Ferreira	2019	Retro	4695	854	3841	70.5	69.2	192	909	261	1266	536	2965	448	2263	54	535
13	SAPPHIRE	2004	RCT	237	117	120	?	?	?	?	?	?	?	?	?	?	?	?
14	CREST	2010	RCT	1181	594	587	69	70	215	191	194	198	524	516	533	535	155	130
15	Yang	2020	Pros	1645	887	758	65.9	64.3	151	130	264	191	622	479	113	112	530	517
	TOTAL			15251	6419	8832	69.06	69.34444444	1637	2060	1778	2447	3689	5363	3047	4278	1696	1589

Outcomes

**Figure 3:**
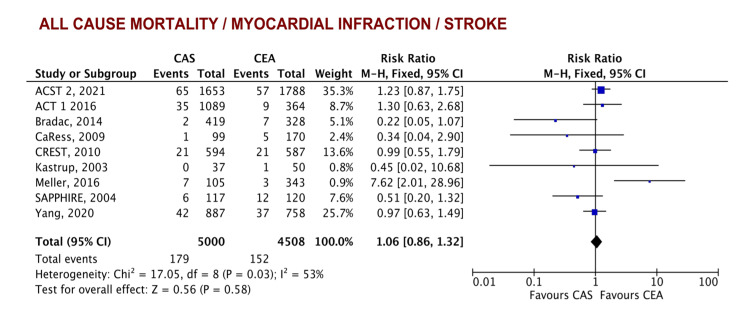
Forest plot of the primary composite outcome of death/any stroke/MI ACST 2 [19], ACT [23], Bradac [28], CaRess [32], CREST [24], Kastrup [31], Meller [26], SAPPHIRE [25], Yang [33] CAS: carotid artery stenting, CEA: carotid artery endarterectomy, MI: myocardial infarction

Primary outcomes: After pooling available results from nine studies, there was no statistical difference in the primary composite outcome of death/stroke/MI (RR = 1.02, 95% CI [0.69-1.51], p = 0.93) (Figure 3).

**Figure 4:**
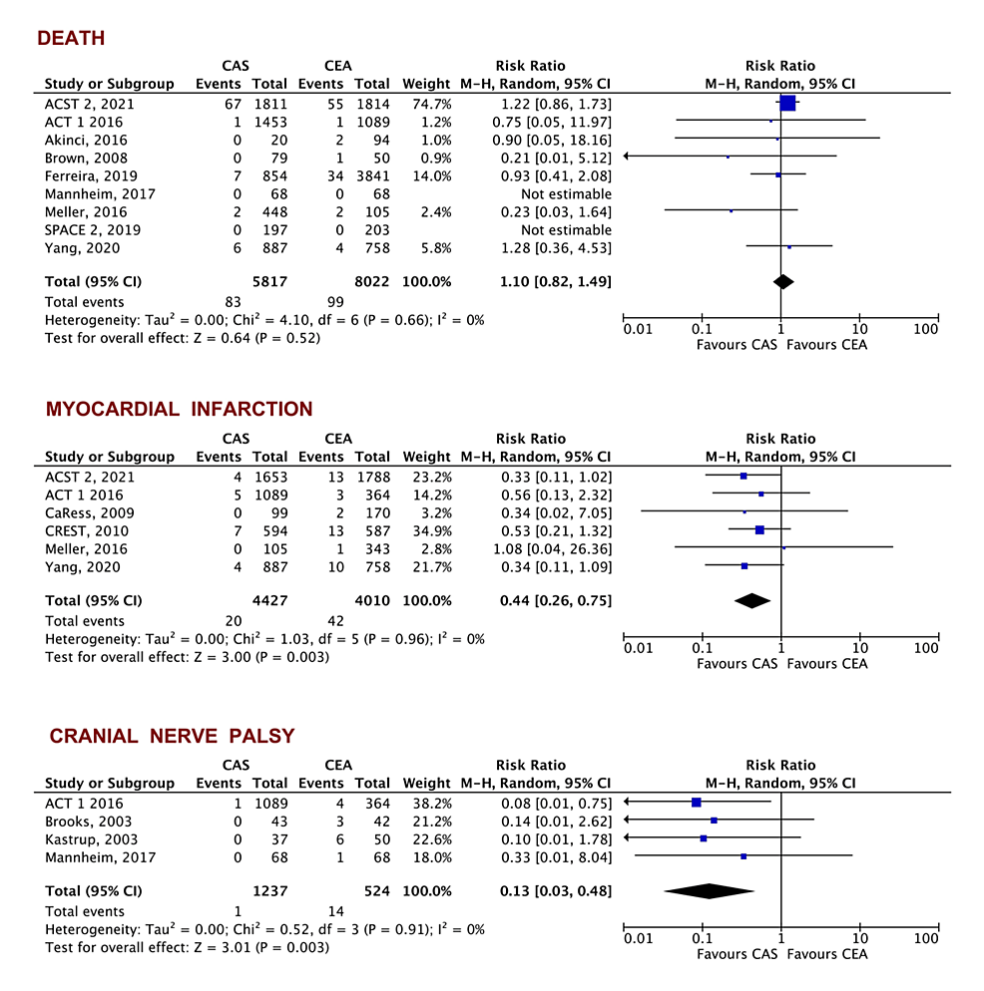
Forest plot of the secondary outcomes of all-cause mortality, MI, and cranial nerve palsy ACST 2 [19], ACT 1 [23], Akinci [29], Brown [27], Ferreira [30], Mannheim [21], Meller [26], SPACE 2 [20], Yang [33], CaRess [32], CREST [24], Brooks [22], Kastrup [31] CAS: carotid artery stenting, CEA: carotid artery endarterectomy, MI: myocardial infarction

**Figure 5:**
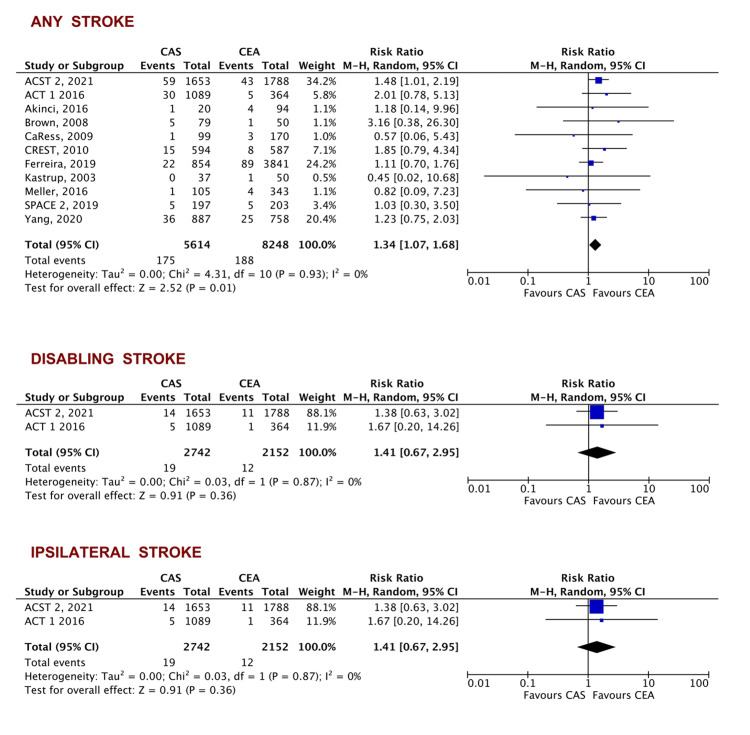
Forest plot of the secondary outcomes of any stroke, disabling stroke, and ipsilateral stroke ACST 2 [19], ACT 1 [23], Akinci [29], Brown [27], Ferreira [30], Mannheim [21], Meller [26], SPACE 2 [20], Yang [33], CaRess [32], CREST [24], Brooks [22], Kastrup [31] CAS: carotid artery stenting, CEA: carotid artery endarterectomy

Secondary outcomes: No difference was found in the secondary outcome of all-cause mortality (RR = 1.10, 95% CI [0.82-1.49], p= 0.52). CAS was associated with a lower risk of MI (RR = 0.44, 95% CI [0.26-0.75], p = 0.003). CAS was associated with a slightly higher risk of stroke (RR = 1.34, 95% CI [1.07-1.68], p = 0.01) with no difference in the occurrence of disabling stroke (RR = 1.41, 95% CI [0.67-2.95], p = 0.91) or ipsilateral stroke (RR = 1.65, 95% CI [0.95-2.89], p = 0.08). The occurrence of cranial nerve palsy was significantly lower with CAS when compared to CEA (RR = 0.13, 95% CI [0.03-0.48], p = 0.003) (Figures 4, 5).

Sensitivity Analysis

Sensitivity analysis performed using fixed effect models for both primary and secondary outcomes revealed similar results.

Discussion

A recent JAMA meta-analysis revealed that the overall incidence of ipsilateral stroke in patients with asymptomatic carotid stenosis (3.2%) is higher than the previously reported rate of 1% [4]. The incidence is even higher (4.3%) in patients with features of high-risk plaques [4]. Current guidelines recommend revascularization as a reasonable approach for stroke prevention in high-risk asymptomatic patients. The best choice of intervention however remains controversial with constant advancement in techniques and the emergence of new data, thus mandating the need to reevaluate revascularization strategies for carotid artery stenosis. We evaluated all the currently available data including the most recent and the largest study comparing the safety of these two interventions [19].

Our analysis revealed no significant statistical difference in the primary composite outcome of death/MI/any periprocedural stroke. CAS offers the benefit of lower risk of MI and cranial nerve palsy as has been demonstrated in other meta-analyses [9,34]. We found a slightly higher albeit statistically significant risk of periprocedural stroke with CAS. In the pooled analysis, the increased risk of stroke was mainly driven by non-disabling stroke. The previous meta-analyses had revealed a non-significant increased risk of stroke with CAS [9,34]. In our analysis, the statistical significance was mainly driven by ASCT 2 which had reported higher event rates in both groups as compared to the other major trials. However, there was no increased risk of ipsilateral stroke or disabling stroke. The ipsilateral strokes are thought to be associated with catheter manipulation and thus are affected by operator experience. This is also the explanation proposed by authors in the Carotid Revascularization Endarterectomy versus Stenting Trial (CREST) trial for their overall low rates of stroke compared to other trials. Studies assessing operator experience have shown that the outcomes with carotid stenting including perioperative stroke and mortality are lower with experienced operators regardless of their specialty [35,36]. The risk of stroke has also been shown to be associated with lesion characteristics. A study by Moore et al. found that the risk was higher in long lesions and/or sequential remote lesions [37]. The authors concluded that the safety of CAS was comparable to CEA in the absence of these high-risk lesion characteristics. In addition, the long-term outcomes have been found to be similar in the two groups in a previous meta-analysis by Moresoli et al. [34]. Jung et al. performed a pooled analysis to compare the long-term durability of the two procedures. They reported that despite the increased peri-procedural risk of stroke, CAS is comparable to CEA in regard to long-term restenosis or occlusion rates [38].

The major strength of our study is that it is thus far the largest and most comprehensive meta-analysis including both real-world data and data from RCTs. Our findings confirmed the previously generally accepted clinical equipoise amongst the two modalities in terms of efficacy in the revascularization of asymptomatic severe carotid stenosis. The superiority of one modality over the other could not be established. Our study had some limitations as well. There was some heterogeneity in the definition of severe asymptomatic stenosis across different studies. Due to a lack of available data, we were unable to account for differences in other possible confounders including operator experience, types of stents used, medications used, and use of embolic protection devices. Long-term outcomes could not be pooled for analysis due to the heterogeneity of the duration of follow-up reported in the different trials. Our analysis is relevant for patients deemed suitable for intervention and did not address the efficacy of medical management alone in the prevention of stroke amongst this population.

## Conclusions

Carotid artery disease is one of the major causes of stroke. CAS and CEA are the two revascularization modalities available for the prevention of stroke in this population. Our analysis demonstrated similar efficacy of the two modalities in terms of the composite end point of death/MI/stroke. CAS is a reasonable alternative to CEA with the additional benefit of decreased risk of MI and cranial nerve palsy. The outcomes of the two interventions are however greatly operator-dependent.
